# Mapping eHealth Education: Review of eHealth Content in Health and Medical Degrees at a Metropolitan Tertiary Institute in Australia

**DOI:** 10.2196/16440

**Published:** 2021-08-19

**Authors:** Melanie Keep, Anna Janssen, Deborah McGregor, Melissa Brunner, Melissa Therese Baysari, Deleana Quinn, Tim Shaw

**Affiliations:** 1 Sydney School of Health Sciences Faculty of Medicine and Health The University of Sydney Camperdown Australia; 2 Research in Implementation Science and eHealth Faculty of Medicine and Health The University of Sydney Camperdown Australia; 3 Charles Perkins Centre Faculty of Medicine and Health The University of Sydney Camperdown Australia

**Keywords:** clinical competence, digital health, educational design, eHealth

## Abstract

**Background:**

With the increasing use of digital technology in society, there is a greater need for health professionals to engage in eHealth-enabled clinical practice. For this, higher education institutions need to suitably prepare graduates of health professional degrees with the capabilities required to practice in eHealth contexts.

**Objective:**

This study aims to understand how eHealth is taught at a major Australian university and the challenges and suggestions for integrating eHealth into allied health, nursing, and medical university curricula.

**Methods:**

Cross-disciplinary subject unit outlines (N=77) were reviewed for eHealth-related content, and interviews and focus groups were conducted with the corresponding subject unit coordinators (n=26). Content analysis was used to identify themes around challenges and opportunities for embedding eHealth in teaching.

**Results:**

There was no evidence of a standardized approach to eHealth teaching across any of the health degrees at the university. Where eHealth content existed, it tended to focus on clinical applications rather than systems and policies, data analysis and knowledge creation, or system and technology implementation. Despite identifying numerous challenges to embedding eHealth in their subjects, unit coordinators expressed enthusiasm for eHealth teaching and were keen to adjust content and learning activities.

**Conclusions:**

Explicit strategies are required to address how eHealth capabilities can be embedded across clinical health degrees. Unit coordinators require support, including access to relevant information, teaching resources, and curriculum mapping, which clearly articulates eHealth capabilities for students across their degrees. Degree-wide conversations and collaboration are required between professional bodes, clinical practice, and universities to overcome the practical and perceived challenges of integrating eHealth in health curricula.

## Introduction

Health and medicine graduates in Australia and internationally are entering increasingly eHealth-enabled work contexts, and eHealth education has been identified as critical for implementing eHealth strategies at national and international levels [1,2]. eHealth refers to the use of information and communication technologies (ICTs) to support health and health care [3]. Such technologies can support clinical and administrative processes, facilitate access to services, and enable health consumers to monitor and manage their own health. Examples include electronic medical records; videoconferencing technology; and wearable devices, such as pedometers and mobile apps; and virtual reality. The introduction of the national My Health Record initiative [4] and the frequency with which people seek health information via web [5] or join web-based support communities [6] further highlight the need for tertiary education to adequately equip students to engage in eHealth-enabled practice. As such, students need a curriculum that enables them to critically analyze the available technologies, implement them in practice where appropriate, and evaluate their effectiveness in specific contexts.

The current literature consistently reports health providers’ lack of confidence in, and knowledge of, using digital technologies as barriers to successful implementation and uptake of eHealth [7-9]. These barriers have also been identified in university students who report being confident in using the technology but not in using the technology for health and health care [10]. Qualitative studies have also highlighted that current tertiary education does not adequately prepare students for working with eHealth tools as clinicians [11,12]. Thus, an eHealth curriculum in higher education needs to focus on how to apply ICT skills in the health context, not just on teaching students how to operate digital tools.

Despite the well-evidenced need to prepare health and medical graduates to work in eHealth contexts, there is limited research exploring how eHealth is currently being taught to students in clinical health and medical degrees. In a pivotal study, Dattakumar et al [13] surveyed coordinators of Australian allied health, nursing, and medical degrees about eHealth education in their curriculum. The researchers found that despite 84% of participants reporting that eHealth was taught, their explanations of the content that was taught showed conflation of eHealth, e-Learning (ie, using the learning management system), and evidence-based practice. Where eHealth content was mentioned, focus was almost exclusively on electronic medical records, with limited content on other key areas of eHealth practice such as telehealth, integration of mobile apps and wearable devices, and data-driven practices. Dattakumar et al [13] concluded that eHealth education at the time was largely informal and inconsistent.

We are now seeing more concerted efforts toward establishing a structured eHealth curriculum that equips and empowers clinical health graduates to work effectively with digital technologies. For example, a recent study by Brunner et al [14] described a capabilities framework of skills and knowledge that is considered key for eHealth practice. The 4 learning domains are related to (1) digital technologies, systems, and policies; (2) clinical practice; (3) data analysis and knowledge creation; and (4) technology implementation and co-design. However, the extent to which these are currently embedded in clinical health curricula is unknown. This study aims to provide an updated and nuanced understanding of how eHealth is currently being taught in health and medicine. Specifically, it aims to determine the extent to which current eHealth teaching at an Australian university maps to the capabilities framework [14], how formally this is integrated into curricula, and teachers’ perceived challenges and opportunities for embedding eHealth into health and medical curricula.

## Methods

### Design

This study used a mixed methods approach, including semistructured interviews and document review. eHealth teaching was mapped across 5 health degrees from a major metropolitan Australian university with approximately 60,000 students: physiotherapy, nursing, dentistry, oral health, and medicine. eHealth teaching refers to any learning outcomes, assessments, or learning activities (eg, discussions, demonstrations, and case studies) about the use of digital technologies in health care. Examples include applying exergaming in physical rehabilitation or discussing how electronic medical record data can be used to improve health services. The mapping process was conducted to understand the shared challenges in teaching eHealth capabilities across a range of health disciplines, with the understanding that each health discipline has a unique approach to teaching and different clinical knowledge and objectives for their graduates.

### Document Review

Degree or course coordinators provided the researchers with access to all the subject or unit of study documents of core units for review (elective units were not reviewed). In total, 77 units of study outlines across the 5 health degrees (dentistry, oral health, medicine, nursing, and physiotherapy) were analyzed. The purpose of the document review was to investigate the extent to which eHealth was represented in the formal curriculum. The formal curriculum consisted of learning outcomes and assessments. The unit of study outline documents were reviewed for eHealth-related content. At a high level, this included any reference to ICT for health, and on a granular level, this included content on how eHealth skills and behaviors were taught or assessed. Any learning outcomes, assessments, or weekly schedules that mentioned technology, eHealth, telehealth, telemedicine, or examples of health technologies were recorded. The topic and topic frequency were recorded against the unit in which they appeared. Data extracted from the unit outlines were categorized as either formal (eHealth was part of the learning outcomes and formally assessed) or semiformal eHealth teaching (mentioned in the unit outline, usually as a lecture topic, but not formally assessed).

### Interviews

A purposeful sample was used to recruit units of study coordinators across the 5 health degrees. A total of 26 coordinators of units within the 5 health degrees (dentistry and oral health, n=10; medicine, n=3; nursing, n=2; and physiotherapy, n=11) agreed to participate in a semistructured interview. Interview questions were chosen to prompt the unit of study coordinators to think about any informal eHealth teaching that was not captured in formal documentation and to understand barriers and enablers to embedding eHealth in their subjects. Participants were provided with a copy of their unit outline at the interview and asked to describe the learning activities that occurred each week in lectures, laboratories, and tutorial classes. Finally, participants were asked to describe the current challenges to embedding eHealth into their subjects and a *blue sky* question, “If resources were not an issue, how would you like to integrate eHealth into your units of study?”

The duration of an interview was, on average, 30 minutes. The interviews were audio recorded, transcribed, and deidentified before analyses. Content analysis of interview data was performed to identify examples of informal eHealth teaching [15]. Interviews underwent an initial reading so that the researchers could familiarize themselves with the content. Transcripts were coded on subsequent readings, and codes were aggregated into broad categories. Exemplar quotes were extracted from the transcripts and grouped under relevant categories. The criteria for formal and semiformal eHealth teaching were applied again, and the criteria for informal teaching were also applied. Informal teaching included, for example, discussions about eHealth, tutorial activities, or other learning activities that were not part of a summative assessment or subject or course learning outcome or described explicitly in any formal course documentation.

### Ethics

Permission to conduct this study was granted by the Human Research Ethics Committee (protocol: 2016/811) of the University of Sydney.

## Results

### Overview

Of the 77 unit outlines reviewed, 30 (39%) included content that could be directly mapped to the eHealth capabilities framework [14]. Although all health degrees had some content related to eHealth in unit outlines, interviews with unit coordinators revealed that much of this content did not translate into specific learning activities that developed eHealth capabilities. As such, incidental eHealth references, which could not be sufficiently mapped to eHealth capabilities, were excluded from study results. Physiotherapy was the only degree in which the majority of its 15 units of study contained some eHealth content (9/15, 60%). Most of this content, however, was embedded within 2 units; the remaining 7 units only had brief mentions of eHealth.

In examining how the teaching content mapped to the 4 domains of the capability framework [14], there was a strong focus on eHealth tools in the *clinical practice and applications* domain (52/64, 81% of eHealth content). There was less focus on *digital technologies, systems, and policies* (10/64, 16%). There was even less content related to *eHealth data analysis and knowledge creation* (8/64, 13%) and *system and technology implementation* (2/64, 0.03%)*.* Note that some activities addressed multiple domains.

### Types of eHealth Content in Health Degree Curriculum

#### Formal

In total, 4 out of the 5 health degrees mapped—dentistry, medicine, nursing, and physiotherapy—had examples of formal eHealth content in the curriculum. Of these degrees, only a small subset of units had formal eHealth content: dentistry (2/8, 25%), medicine (2/47, 4%), nursing (2/32, 6%), and physiotherapy (5/32, 16%). All examples of formal eHealth content were in the form of unit objectives. Only one unit of study, in the physiotherapy degree, used the term *eHealth*; other degrees either used the term *technology* or did not explicitly refer to eHealth in any form. There were no instances where eHealth capabilities were formally assessed across any of the health degrees.

#### Semiformal

In total, 3 out of the 5 health degrees mapped—dentistry, nursing, and physiotherapy—had examples of semiformal eHealth content in their unit of study outlines. As was the case with formal eHealth content, this semiformal content was only present in a small number of units within each degree. A total of 6 nursing (6/32, 19%) and 5 dentistry (5/8, 63%) units of study had high-level statements relating to developing eHealth relevant knowledge and skills.

#### Informal

All degrees mapped had some examples of informal eHealth teaching; however, the extent of teaching and delivery approaches varied widely. Most degrees had some level of informal eHealth teaching related to ethical or professional use of technology, particularly social media. In more sophisticated examples of informal eHealth teaching, unit coordinators described concrete examples of tutorials where eHealth concepts were discussed. However, it was more common for informal eHealth teaching to be unstructured or implicit:

I think it’s just we try and include, without thinking about eHealth specifically, we try and include stuff in there that’s moving with the times if you like, is more of how I think of it.

Physiotherapy 9

Many coordinators reported a sense that students would pick up eHealth skills over time because of their high level of technology use in everyday life. Unit coordinators also tended to report a belief that informal eHealth teaching was being delivered to the students throughout their degree, but they were unable to give concrete examples of when and how this was happening. In medicine, this tended to occur during clinical placement, rather than in academic units:

Yeah, it [my experience with telehealth] was totally...circumstantial [because of my speciality], but I figured that that was cool. I actually didn’t realise, you know, you hear about it and then okay, well this actually works pretty well.

Medicine 1

### Challenges to Embedding eHealth Teaching

#### Perceived Relevance

The relevance of eHealth teaching was frequently cited as a challenge in implementing it in health curricula. Different aspects of relevance were highlighted by participants, including the relevance of teaching eHealth to already *tech-savvy learners* who potentially use digital tools every day, the relevance of learning about eHealth in isolation outside the clinical context, and a lack of perceived value for teaching students about eHealth tools without immediate practice application. For example, one participant stated:

The students didn’t particularly like it [tutorial focused on health apps], it was kind of this just tute that stood out as this different thing [from other physiotherapy content] and it was just apps...we didn’t get very good feedback.

Physiotherapy 8

Some participants also attributed their doubt about the relevance of eHealth, given the variable use of digital technologies across the health sector. A perceived lack of actual uptake of digital tools in clinical practice was considered a challenge when attempting to convey relevance to learners. For example:

Teaching hospitals, they’re still using paper records, paper files, everything’s done on paper...So until those go digital, it’s pointless talking about having graduates who are IT ready.

Oral health 3

This disconnect between clinical practice and classroom experiences was a strong subtheme within perceived relevance, which is a challenge for eHealth teaching. Some interviewees felt that even when eHealth teaching was embedded in health units, the learnings did not align with what students encountered during clinical placements or when they entered practice.

As observed in the following quote, one participant questioned the limitations and appropriateness of eHealth-enabled practice for aspects of clinical care more traditionally delivered face to face and with hands-on methods. Such quotes clearly captured participants’ attitudes toward eHealth methods:

...Our assessment has a lot more of a physical focus where you’re actually watching the patient move and looking for the impairments and looking for the adaptive strategies and thinking about why they might be moving that way and what you can do about it. Technology’s a little bit limited with that without going to really fancy stuff, which is more used for research rather than clinically anyway. You know, 3D motion capture...[Physiotherapy 9]

Participants considered it a major challenge that they could not comprehensively showcase eHealth tools because of access to, and licensing restrictions for, commercial products commonly used in clinical practice. Being very systems focused, some participants expressed that the use of commercial eHealth products presented a challenge for teaching because products either changed too frequently or showcasing one over another could be viewed as staff endorsement of a product. Hence, some interviewees indicated that it was more appropriate to teach eHealth in the workplace rather than at university. One interviewee commented:

You learn it on your first job, when your practice manager, sits you down and says, “Look, this is our software and this is how we use it.”

Oral health 3

Finally, there was a lack of alignment in terms of the core eHealth competencies participants considered essential to graduates. There were different priorities for eHealth competencies in different health specialties. For example, within physiotherapy, learning system data entry was not perceived as high a priority as learning the value of quality data and how it can be used to improve care. In contrast, within medicine, more emphasis was placed on the use of data systems, such as electronic medical records and informatics skills.

#### Students’ Inexperience as Clinicians

Participants consistently emphasized that students first needed to develop their clinical reasoning before they could engage with eHealth, which was largely considered to be more advanced. Participants reported that students, even in the second year, still have misconceptions and do not understand their own practice well enough to be using eHealth technologies, such as videoconferencing, in their practice.

One participant stressed the importance of emphasizing eHealth in the context of patient safety. As exemplified in the following quote, this participant considered students’ confidence and high level of digital literacy to be a challenge when matched with limited clinical skills:

The big thing is because our students are tech savvy, they’re not afraid of technology which is really tricky for us...They won’t think twice about touching a touch screen on a ventilator because they grew up with screens. To them a screen is not a scary thing. There’s a whole patient safety aspect to the technology that we have to really look at.

Nursing 1

One participant expressed doubt related to students’ confidence and capacity to express concerns when eHealth is being inappropriately used, which could be attributed to a lack of perceived experience in the clinical context. It also highlighted a disparity between poor use of digital technologies in clinical practice and examples of best practice of eHealth in classes, which can be challenging to overcome in eHealth teaching:

Talking in one of my last tute’s...They were saying that doctors text results to their patients which is an unsecure source...You probably don’t have the capacity to tell the GP you work for that he or she shouldn’t be texting results through the phones.

Nursing 2

#### Educators’ Inexperience With eHealth

A number of interviewees across each of the health disciplines indicated their own inexperience with eHealth as a challenge to embedding it. They raised questions about the quality of apps currently available and reported a limited understanding of the current research on high-quality, eHealth-enabled care. Others also emphasized that they felt students’ experience with technology outstripped their own, which made it challenging to know how to approach teaching eHealth. One interviewee commented:

I think our students our very savvy...So I feel that academics, we’re the ones that need the assistance, I don’t think we necessarily need to teach our students that [eHealth] because I feel that they already know that, they’re happy to research that, in fact that’s the one thing they do like you know.

Oral health 2

Some participants suggested that they had experience teaching some areas of eHealth, such as telehealth, use of apps, or exergames (games that are used to promote exercise) as adjuncts to face-to-face care, but lacked tools to teach eHealth holistically in a manner that encompassed a breadth of technologies. In addition, one interviewee noted that it was just generally challenging to teach eHealth because digital technologies, their applications, and policies around their use were frequently changing.

#### Practical Challenges

Interviewees identified several practical challenges impacting eHealth teaching and curriculum. Crowded curriculum, limited resources, time and effort, and alignment of a curriculum with accreditation requirements were the issues that were raised. For example, one participant cited time within classes and the curriculum more broadly as limitations to embedding eHealth within classes:

...we cannot put much in these...our tutorials are one hour tutorials only...And in the one hour, because we know that the physio curriculum is constrained by accreditation requirements as well, so are there particular manual things that the students have to practise in that one hour?

Physiotherapy 1

However, all participants were supportive of exploring creative ways of integrating eHealth ideas within essential areas of curriculum focus, for example, integrating eHealth methods into a tutorial where learning outcomes are focused primarily on a clinical intervention, such as pain management or cardiopulmonary rehabilitation.

Limited resources include lack of access to appropriate tools or databases, which is explicitly linked to a lack of funding for hardware and software, such as licenses for apps, exergaming and virtual reality equipment, and simulated electronic medical records. Where access to these tools may be available, one participant stated that it was time consuming to interact with the tools to design meaningful learning activities:

I tried last year to set up dummy accounts for Fitbits and things and picked a couple of students to take one and have a go, and then we’d have a look at their data so they could see how they would access that data from the thing. It took me over half a day setting up because you have to set it all up through an e-mail account. I set up all these student Google Gmail accounts and then had to prime the Fitbit and then do whatever.

Physiotherapy 5

### Suggestions for eHealth Teaching in the Future

Although embedding eHealth in the health curriculum posed a number of challenges, interviewees also saw many opportunities for integrating eHealth in the future. Participants were uncertain about the extent to which eHealth teaching should be embedded into existing units of study or taught in stand-alone units. Analyses suggested that, of the health degrees that currently had a higher frequency of eHealth topics, support for taking an embedded teaching approach, rather than stand-alone eHealth units, was more common. In degrees that had less eHealth teaching, participants acknowledged that future efforts need to focus on blending eHealth into the curriculum, rather than keeping it as a stand-alone area. It was also suggested that greater effort was needed to use digital technology to deliver the curriculum, rather than just teaching about the use of technology.

Interviewees suggested a range of methods for incorporating eHealth teaching into health curricula, including didactic content, course readings, and embedding eHealth scenarios into assessments (eg, rural case study). Other suggestions included more interactive and hands-on methods, including tutorials built around eHealth case studies and role plays, and live demonstrations of digital technologies by current practitioners.

In addition, interviewees identified a number of opportunities to embed eHealth in the curriculum in a way that could build practical eHealth skills. Examples included providing learners with opportunities to design and build eHealth tools, using current, in-the-market digital technologies such as apps or virtual reality products and exergames, and analyzing hospital data as a manager:

I suppose what we are missing...[is] using health data. As you’ve seen I talk about that in the presentation in terms of how if you’re the manager of a hospital physio department you’re making decisions about staffing, about prioritising services based on clinical data that’s been collected in your hospital and summaries, and what have you. Whether I should get the students to go maybe set them a challenge or something to go and look at...I don’t know...You can go into the Bureau of Health Information’s webpage, and you can generate reports and things.

Physiotherapy 5

## Discussion

### Principal Findings

This mixed methods study reviewed the unit of study documentation and conducted interviews with unit coordinators to determine the extent to which current eHealth teaching at an Australian university maps onto the eHealth capabilities framework [14]. In addition, the study explored the types of learning activities (formal, semiformal, and informal) used, challenges that unit coordinators experience in embedding eHealth content in health curricula, and their suggestions for improving eHealth teaching in their units. Of the 4 eHealth domains in the capabilities framework [14], learning activities in dentistry, oral health, medicine, nursing, and physiotherapy tended to focus on clinical practice and applications. There were some examples of learning activities in digital technologies, systems, and policies and data analysis and knowledge creation, and there are very few learning activities in the system and technology implementation domain. Interestingly, only the medical and nursing curricula included activities related to system and technology implementation. This may reflect differences in the types of health services that medicine and nursing graduates work in (eg, hospitals where consideration of a system approach is more prominent) compared with physiotherapy and dentistry graduates. Most examples of eHealth learning were informal. Unit coordinators also identified perceived relevance, students’ inexperience as clinicians, teachers’ inexperience with eHealth, and practical challenges of a crowded curriculum and limited resourcing as hurdles for embedding eHealth into health curricula.

This study builds on the existing literature on eHealth curricula [13,16,17] by evaluating the formal, semiformal, and informal curricula of several health degrees. Although only 1 unit used the term *eHealth* in formal unit outlines, the term was included in some learning outcomes but not in any of the summative assessments. There were fewer semiformal eHealth learning activities across the degrees and individual units. Informal learning was present, but the approaches used varied greatly. The limited adoption of eHealth in clinical practice [18-20] seems to be reflected in the small number of formal and semiformal eHealth learning activities in the sample included in this study. When mapped against the eHealth capabilities framework [14], learning activities tended to focus on clinical practice and applications with far fewer examples of digital technologies, systems and policies, data analysis and knowledge creation, and system and technology implementation. Given that assessment drives learning [21], the findings of this study highlight the need for a deliberate assessment of learning outcomes that explicitly focus on eHealth capabilities, particularly around system and technology implementation.

Consistent with previous literature [22], the findings in this study showed that despite interest in including eHealth content in health curricula, unit coordinators have identified several challenges for doing so. These include perceived relevance (from the students’ perspective), educators’ inexperience with incorporating eHealth teaching into curricula, students’ inexperience as clinicians, and disconnect between classroom experiences and clinical practice. Together, these challenges suggest an opportunity for degree-wide conversations about when and how to introduce students to eHealth. Both learning and teaching theory and research highlight the importance of perceived relevance in motivating students to engage with content. For example, the attention, relevance, confidence, and satisfaction model by Keller [23,24] states that relevance provides 1 of the 4 conditions (the others being attention, confidence, and satisfaction) under which motivation can occur in the learning context. Relevance refers to the extent to which learning addresses students’ personal needs for learning it. Therefore, in the case of students in clinical health degrees, this personal learning need is likely the extent to which the information or skills they are being taught enables them to be a better practitioner. Therefore, effective eHealth education needs to be taught in a way that is perceived as relevant and core to the students’ professional practice.

Given that some unit coordinators consider eHealth as part of an emerging best practice, conversations about curriculum should also include professional and accrediting bodies. This coordinated approach would enable subjects taught in earlier years of the curriculum to introduce eHealth concepts while continuing their focus on students’ development as clinicians and then scaffold students into more advanced eHealth practice in later years. Discussions about best practices can also be used to inform future iterations of accreditation requirements, which can, in turn, help address concerns around crowded curricula.

The challenge of a crowded curriculum is not unique to eHealth. With accreditation and registration requirements, health and medicine curricula are at capacity with mandatory content, leading to challenges in integrating Aboriginal and Torres Strait Islander education [25] and more comprehensive patient safety education [26] into the medical curriculum.

The practical challenges of introducing more content into health and medical education are coupled with pedagogical considerations. For example, traditional health education uses a building block approach. The curriculum consists of foundational units of study where students learn specific content or skills, such as biology or research methods, in separate subjects. This knowledge is then brought together in more complex and applied scenarios in senior years through more profession-specific units or practicum experiences. Students who study in this context report a lack of understanding about how these foundational units fit into their professional development and can perceive the units as less relevant or integral to the *core* curriculum [27].

Unit coordinators also highlighted their own inexperience with eHealth as a potential barrier to including content in their curricula. Part of this could also be related to perceived relevance and current misconceptions about eHealth (conflating it with e-Learning or evidence-based practice), as documented in the literature [13]. Given the limited learning activities generally and inconsistent mapping to the eHealth capabilities framework, there is a need for targeted professional development and at-elbow support for teachers. This would also include understanding how eHealth is currently applied in clinical practice and designing learning activities to explicitly promote this relevance.

Despite these challenges, there was general enthusiasm and keenness for introducing eHealth. Unit coordinators suggested a range of interactive activities designed to promote eHealth capabilities and critical thinking. However, there was continued uncertainty regarding how best to deliver eHealth education more broadly. One approach is to embed eHealth teaching directly into units of study that are central to health curricula, which can ensure clear links between clinical learning and technological learning. Alternatively, stand-alone units that explore eHealth can be used. These enable deep dives into eHealth knowledge and have the potential to more easily align with comprehensive eHealth frameworks [14]. However, such units continue to silo eHealth education away from core health training, which limits the reach of the content to all health graduates.

### Limitations

This study provides a foundation for understanding and improving the way eHealth is included in health curricula. One limitation, however, is the unequal representation of staff from across the 5 degrees. Owing to inconsistent administration or unit organization practices, it was difficult to identify the person responsible for every learning module or unit of study. Physiotherapy used defined units of study with clear core and elective units. Uptake of participants was also greater in physiotherapy; therefore, there were more participants from that degree than from nursing, oral health, dentistry, and medicine. Despite this, the themes that emerged from the content analysis of interviews were consistent, and no new ideas were introduced. This suggests data saturation.

### Implications and Future Directions

Future research should consider mapping eHealth teaching in health degrees that may have different accreditation requirements, such as more generalized health and science degrees. Graduates from these degrees often go onto corporate roles in health and can be influential in health systems and technology management and implementation.

This study undertook a high-level mapping process to understand shared challenges in eHealth teaching across health degrees. Future researchers may wish to conduct degree-specific research to understand the unique challenges faced by eHealth educators in individual health degrees. The findings from this study suggest that there may be disciplinary differences in eHealth focus that may reflect differences in the types of workplaces and work that graduates from different health degrees enter. For example, medicine and nursing were the only degrees that currently included content relating to system and technology implementation. This may reflect the greater proportion of graduates entering hospitals and the emphasis on these skills in that setting. Further research exploring disciplinary differences in eHealth focus is also needed.

### Conclusions

At present, there is limited inclusion of eHealth in health and medical curricula. This includes formal (learning outcomes and summative assessments), semiformal (documented in unit outlines but not formally assessed), and informal (not documented but explicitly taught in classes) learning activities. Where there was eHealth content, it tended to focus on clinical applications rather than systems and policies, data analysis and knowledge creation, or system and technology implementation. There is a need for more explicit strategies for embedding eHealth capabilities across health and medical degrees. We recommend degree-wide conversations and collaboration between professional bodes, clinical practice, and universities to overcome the practical and perceived challenges of integrating eHealth in health curricula. Overall, unit coordinators were supportive of including eHealth in their teaching and welcoming of opportunities to learn how to do so. Future research could build on this to develop and evaluate examples of best practices in eHealth curricula.

## Abbreviations

ICT: information and communication technology
